# Supplementary material for: Biochemical and Genomic Characterization of the Cypermethrin-Degrading and Biosurfactant-Producing Bacterial Strains Isolated from Marine Sediments of the Chilean Northern Patagonia
Source: Mar Drugs. 2020 May 13;18(5):252. doi: 10.3390/md18050252 (PMC7281626; doi:10.3390/md18050252)
Supplement: Supplementary file 1 [file marinedrugs-18-00252-s001.pdf]

Table S1. Phenotypic and biochemical characteristics of cypermethrin-degrading and biosurfactant-producing bacterial strains isolated from Northern Chilean Patagonia.

Strain	MS1	MS4	MS8	MS10	MS11	MS12	MS13**	MS14	MS15a**	MS16**	MS19**
**Gram stain**	+	-	-	-	+	+	+	+	-	+	-
**Cell morphology**	cocci	bacilli	rod	rod	bacilli	bacilli	rod	rod	bacilli	bacilli	bacilli
**Motility**	-	+	+	+	-	-	-	-	+	-	+
**Catalase**	+	+	+	+	+	+	+	+	+	+	+
**Cypermethrin* growth 50 mg L^{-1} (OD600 nm)**	+	+	+	+	+	+	+++	+	+++	+++	+++
**E_{24} (%)***	12	94	76	32	24	82	77	38	60	79	71

* Cells grown on TSA at 28 °C for 24 h.
** strains selected for genomic analysis.

Table S2. Identification by comparative sequence analyses of cypermethrin-degrading and biosurfactant-producing bacterial strains isolated from Northern Chilean Patagonia.

Strain	Accession number	Closest type strain (Accession N°)	Score (% Identity)
MS1	MK271075	*Rhodococcus erythropolis* ATCC 4277 (AY281111.1)	435/436 (98)
MS4	MK271077	*Serratia odorifera* DSM 4582 (NR_114578.1)	702/730 (96)

MS8	MK271079	*Pseudomonas oryzihabitans* LMG7040 (NR_117269.1)	729/733 (99)
MS10	MK271080	*Pseudomonas* sp. DSM 12280 (AM088476.1)	700/740 (95)
MS11	MK271081	*Rhodococcus globerulus* DSM 43954 (NR_026184.1)	716/718 (99)
MS12	MK271082	*Rhodococcus globerulus* DSM 43954 (NR_026184.1)	707/709 (99)
MS13	MK271083	*Rhodococcus* sp. DSM43943 (X80616.1)	702/720 (98)
MS14	MK271084	*Rhodococcus* sp. DSM43943 (X80616.1)	696/713 (98)
MS15	MK271085	*Pseudomonas* sp. DSM 12280 (AM088476.1)	703/742 (95)
MS16	MK271086	*Rhodococcus globerula* DSM 43954 (NR_026184.1)	704/706 (99)
MS19	MK271087	*Pseudomonas marincola* KMM 3042 (NR_041592.1)	707/711 (99)

M.S.: Marine Sediment
